# Mutual synchronization of spin torque nano-oscillators through a long-range and tunable electrical coupling scheme

**DOI:** 10.1038/ncomms15825

**Published:** 2017-06-12

**Authors:** R. Lebrun, S. Tsunegi, P. Bortolotti, H. Kubota, A. S. Jenkins, M. Romera, K. Yakushiji, A. Fukushima, J. Grollier, S. Yuasa, V. Cros

**Affiliations:** 1 Unité Mixte de Physique CNRS, Thales, Université Paris-Sud, Université Paris-Saclay, Palaiseau 91767, France; 2 Spintronics Research Center, National Institute of Advanced Industrial Science and Technology (AIST), Tsukuba, Ibaraki 305-8568, Japan

## Abstract

The concept of spin-torque-driven high-frequency magnetization dynamics, allows the potential construction of complex networks of non-linear dynamical nanoscale systems, combining the field of spintronics and the study of non-linear systems. In the few previous demonstrations of synchronization of several spin-torque oscillators, the short-range nature of the magnetic coupling that was used has largely hampered a complete control of the synchronization process. Here we demonstrate the successful mutual synchronization of two spin-torque oscillators with a large separation distance through their long range self-emitted microwave currents. This leads to a strong improvement of both the emitted power and the linewidth. The full control of the synchronized state is achieved at the nanoscale through two active spin transfer torques, but also externally through an electrical delay line. These additional levels of control of the synchronization capability provide a new approach to develop spin-torque oscillator-based nanoscale microwave-devices going from microwave-sources to bio-inspired networks.

## Abstract (web summary)

The spintronics based complex network is promising for next generation computing systems but hampered by short-range spin-wave coupling. The authors make progress by achieving long range and tunable mutual synchronization of two spin-torque oscillators with improved emission power and signal linewidth.

In 2005, two seminal papers by S. Kaka *et al*.^1^ and F.B. Mancoff *et al*.^2^, successfully described the synchronization by spin wave coupling between two closely-spaced spin torque oscillators (STOs). Owing to the intrinsic short-range nature of the spin-wave coupling (typically around 1 μm in metallic magnetic materials such as NiFe), a persistent goal in the last decade has been to achieve an in-depth understanding of the synchronization process to control it. The efficiency of the synchronization is indeed a crucial point to further increase substantially the number of synchronized oscillators. Thanks to continuous research efforts^3^,^4^,^5^,^6^,^7^,^8^, new demonstrations of synchronization through dipolar^9^ or spin-wave^10^ coupling have been achieved in the last couple of months. However, alternatives to these short-range magnetic couplings, that are only efficient over the spin-wave decay length or decay length of dipolar fields (both of around 1 μm (refs 9, 10)), must be found to achieve large and controllable arrays of synchronized spin torque oscillators. With this perspective in mind, the long-range electrical coupling, that we theoretically proposed in 2006 (ref. 11), represents probably the most promising approach. Beyond the importance of synchronizing arrays of oscillators for applications, it is also anticipated that spin-torque oscillators become a table-top model implementation^12^,^13^ of the underlying physics of non-linear phenomena, for example, total, partial or chaotic synchronization in arrays composed of nanoscale dynamical systems^14^,^15^.

In the following, we demonstrate experimentally that the self-emitted radiofrequency current is an efficient source of coupling for achieving the mutual synchronization of spin torque oscillators, either closely or distantly separated (through a few meters of cables). Moreover, we show a quantitative agreement with the predicted improvements in terms of both emitted power and spectral coherence in the synchronized state contrary to all previous studies based on short-range coupling. More interestingly, owing to the nature of this coupling mechanism, we achieve a fine control of the different microwave-features of the synchronized state (frequency, power, synchronization range and the phase shift between the oscillators) at the nanoscale. We highlight two key parameters to control the synchronized state: the intrinsic nonlinear parameters of the oscillators and, more originally, the ratio between the two active components of spin transfer torques, that is, Slonczewski-like (SL) Torque and Field-like (FL) Torque. In addition, we also demonstrate that we can tune the long-range coupling through an electrical delay line. The full control of the synchronization capability allows the enhancement of the usually observed low power and poor spectral coherence of these oscillators, which is vitally important for the development of nanoscale microwave devices. This should also allow fine-tuning of the coupling constant between each oscillator inside a network, a crucial step for mimicking basic functionalities of the brain^12^,^16^,^17^,^18^ in nanoscale bio-inspired devices.

## Results

### Mutual synchronization through self-emitted microwave currents

**Figure 1:**
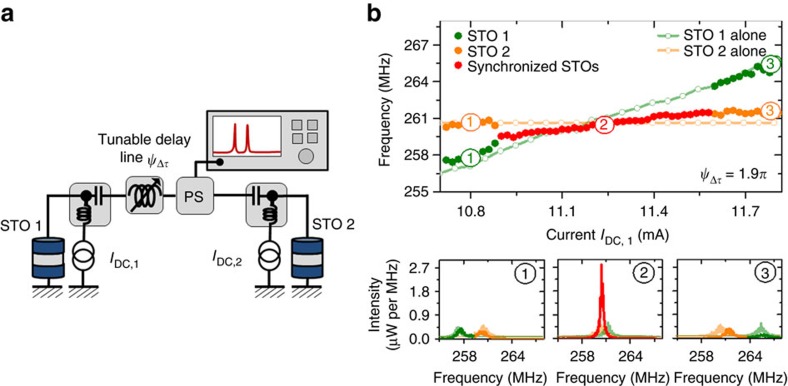
Mutual synchronization of two spin torque oscillators. (**a**) Scheme of the electrical circuit for the mutual synchronization of two oscillators independently supplied by two currents and connected through the microwave port of two bias tees with a tunable delay line *ψ*_Δ*τ*_. The detected signal is measured using a spectrum analyzer, connected to the delay with a −6 dBm power splitter (PS). (**b**) Evolution of the frequency of the interacting STOs as a function of *I*_DC,1_ while *I*_DC,2_ is fixed to +10.6 mA. Corresponding spectra for *I*_DC,1_=+10.8 mA (1), +11.25 mA (2), +11.8 mA (3) (Non-interacting oscillator properties when one is switched off are in orange and green softened curves).

The main purpose of this study is to investigate the electrical coupling between two widely spaced STOs as a possible source of interaction to reach an optimal state of in-phase and coherently mutually synchronized STOs. To reach this goal, two types of electrical connection between the STOs might be envisaged, namely either a connection in series or in parallel, even though the physical mechanisms responsible for the synchronization will be the same in both cases^11^,^19^,^20^. In the experiments presented here, we will consider the latter case of a parallel connection between the two spin torque oscillators. As shown in Fig. 1, each STO is independently supplied by a DC current source, allowing them to enter independently in a regime of sustained oscillations through the action of the spin torque. The microwave signal emitted by both oscillators is the coupling mechanism driving the mutual synchronization process, achieved by having the microwave ports of the two bias tees electrically connected through microwave cables and a tunable delay line (Fig. 1). Finally, we insert a power splitter (PS) in the circuit to record the output microwave signal originating from the two STOs using a spectrum analyzer.

An essential condition for the experimental observation of the mutual synchronization between STOs is that the synchronizing force has to be larger than the thermal fluctuations^15^. In addition, one also requires STOs with a narrow linewidth and a large output power together with an efficient injection locking process to an external microwave current. These requirements motivated our choice to work with vortex based STOs, that we have extensively studied in the past and which have the required microwave properties. Note however that there are no intrinsic difficulties that similar mutual synchronization might be achieved using other types of STOs operating at higher frequencies. Here we use STOs based on the spin transfer induced dynamics of two interacting vortices in a spin-valve located above a magnetic tunnel junction. From our previous studies^21^,^22^,^23^, we have demonstrated precisely the vortex configurations and the spin torque components (the ones associated to the vortex-like spin polarization) that result in a sustained dynamical state at room temperature showing the required oscillator properties, that is, a strongly coherent (∼100 kHz) and powerful (∼400 nW) emitted radio frequency signal^22^.

As pointed out already, the electrical coupling mechanism allows us to easily access the dynamical properties of each oscillator when they are interacting or when they are independent (which is not the case for short-range coupling with the noticeable exception of the study by Kaka *et al*.^1^). This is a crucial point as it provides a unique opportunity to characterize properly the microwave properties of the synchronized state by comparing the signal recorded on the spectrum analyzer in two independent measurements. A first set of measurements is recorded when the two STOs are self-oscillating due to the spin transfer torque (the two DC sources supplying the STOs are switched ON, Fig. 1a). For that, we keep the applied DC current constant on STO2 and sweep the current applied on STO1. Note that these measurements have been recorded for an optimized electrical delay length 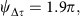 (expressed in period of STO's oscillations); the influence associated with a change of the delay will be discussed in the last section of this article. In Fig. 1b, we display the first experimental evidence of mutual synchronization between STOs via electrical coupling. Indeed, in region 2 (red dots in Fig. 1b) we observe a single peak having a much larger power than the two peaks outside the synchronization bandwidth (region 1 and 3 with green and orange dots in Fig. 1b). This single peak in region 2, where the two STOs have a common frequency, is observed over a frequency range equal to 2 MHz which corresponds to the synchronization bandwidth Δ*ω*_sync_.

In a second set of measurements, we have recorded independently the microwave signal from each STO while the current supplied to the other STO is zero. Thus we can compare quantitatively the emitted signals in the interacting and non-interacting states. Data corresponding to STO1 (*I*_DC,1_ is ON, *I*_DC,2_ is OFF) and STO 2 (*I*_DC,2_ in ON, *I*_DC,1_ is OFF) measured independently are respectively shown in light green and in light orange in Fig. 1b. A more complete characterization of the individual STOs is presented in the Supplementary Note 1. In the region of mutual synchronization (region 2 on Fig. 1b), we notice that the emitted signal is much larger than for the non-interacting STOs. The frequency of the synchronized state also differs from the frequencies of the two non-interacting STOs indicating that the synchronization process is not unidirectional and that the two STOs are mutually synchronized. Furthermore, the spectral coherence in this region 2 is also increased compared to the non-synchronized state as shown in the bottom graphs of Fig. 1b.

### Microwave properties in the synchronized state

**Figure 2:**
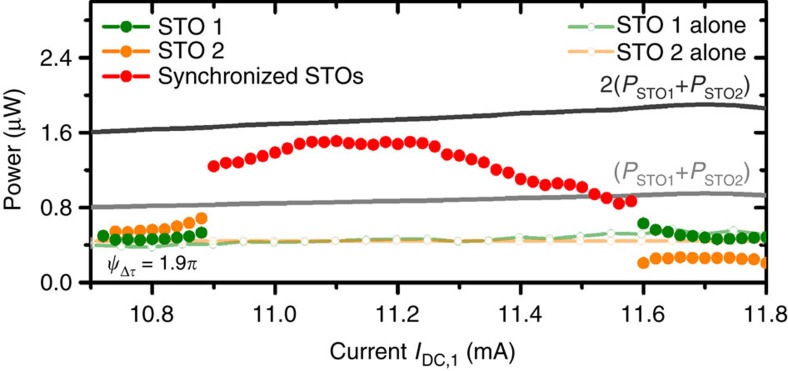
Emitted microwave power depending on the magnitude of the DC current injected in the oscillator STO 1 (*I*_DC,2_ is fixed to +10.6 mA for STO 2). The characteristics of each STO when one is switched off are shown in green and orange softened curves.

**Figure 3:**
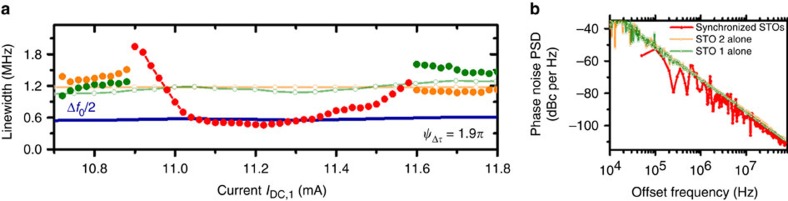
Spectral coherence in the synchronized state. (**a**) Emitted spectral linewidth depending on the magnitude of the DC current injected in the oscillator STO 1 (*I*_DC,2_ is fixed to +10.6 mA for STO 2). (**b**) Phase noise power spectral density in the synchronized state (*I*_DC,2_ is fixed to +10.6 mA and *I*_DC,1_ is fixed to +11 mA) and in the non-interacting states. The characteristics of each STO when one is switched off are shown in green and orange softened curves).

To analyse quantitatively the microwave features of the mutually synchronized STOs, we evaluate the emitted power (Fig. 2) and the spectral coherence (see Fig. 3) in the synchronized state as well as in the non-interacting states. First we notice that the two non-synchronized oscillators have very similar power amplitude (*P*_STO1_–*P*_STO2_) and linewidths (Δ*f*_STO1_–Δ*f*_STO2_) (see light green and orange dashed line in Figs 2 and 3). Given these similarities, we can consider that the two STOs are almost identical to the exception of their frequency difference. Thus, we can easily compare the properties of the synchronized state to the theoretical predictions made with similar hypothesis. It then results that their respective emitted powers and linewidths are close to their average mean value *P*_0_ and Δ*f*_0_.

In Fig. 2, we focus on the emitted power and observe a maximum of power close to the center of the synchronization bandwidth Δ*ω*_sync_, and minima at the edges. From these features, we can draw two important conclusions. First, the measured power in the synchronized regime is superior to 2*P*_0_, the sum of the emitted power of the two non-interacting STOs. This experimental observation demonstrates per se that we really achieve the mutual synchronization of the two STOs (and not a master-slave coupled dynamics). In the best experimental conditions, that is, the center of the synchronized regime, we find that the total emitted power *P*_tot_ reaches almost 4*P*_0_, up to 1.6 μW with the two STOs measured here. We emphasize that such strong and quantitative power enhancement in the synchronized state at zero frequency detuning is theoretically expected but has never been observed until now. Note that in Kaka *et al*.^1^and Rippard *et al*.^3^, the observed increase of emitted power when the two nanocontact STOs were synchronized has been compared to the power of each single STO in the synchronized state, but not directly compared with the emitted powers measured in the non-interacting states. Thus our observation represents a crucial advance for the further improvements of the emitted power of nanoscale STOs^12^, which until now has represented an important roadblock for a range of proposed STO-based applications.

In parallel to the strong increase of the emitted power in the synchronized state, we also find a remarkable improvement of the spectral coherence^1^,^2^,^5^,^6^. As shown in Fig. 3a, the linewidth of the synchronized peak at the center of the synchronization bandwidth is reduced down to 550 kHz, corresponding to a reduction by a factor of two compared with the non-synchronized states. Such a significant reduction provides a clear confirmation that the phase noise in the synchronized state is driven by the diffusion of the phase sum (ref. 15 and Supplementary Note 2). This quantitative agreement between the linewidth in the synchronized and non-interacting states indicates the coherence of the synchronization state. Such a quantitative comparison between the synchronized and the non-interacting states can be obtained only for a robust synchronization process, that is, with a small number of desynchronization events during the acquisition time. In Fig. 3, we plot the power spectral densities of the phase noise (extracted by time domain measurements^23^) in the synchronized and non-interacting states. To extract the phase noise in the synchronized state, the time trace should not present any desynchronization events or phase slips^23^. If this is the case, it is possible to describe entirely and uniquely the phase dynamics of the two STOs by the properties of the synchronized state. In Fig. 3, the plotted phase noise corresponds to a time trace of 30 μs meaning that there is no desynchronization event for more than 7,500 periods of oscillations.

The synchronized state is much less stable at the edges of the synchronization bandwidth. In these regions, the measured linewidths are larger, and can even be larger than that of the independent STOs. This strong enhancement of the linewidth is most probably associated with either frequency pulling and/or phase slips resulting in temporary loss of the synchronized state^9^,^23^. To our knowledge, our report is the first quantitative confirmation that both the spectral coherence and emitted power in the synchronized state of N-synchronized STOs, whatever the coupling mechanism, can be respectively enhanced by a factor N (refs 15, 24) and N^2^ (ref. 11).

### Control of the synchronized state through a tunable delay

**Figure 4:**
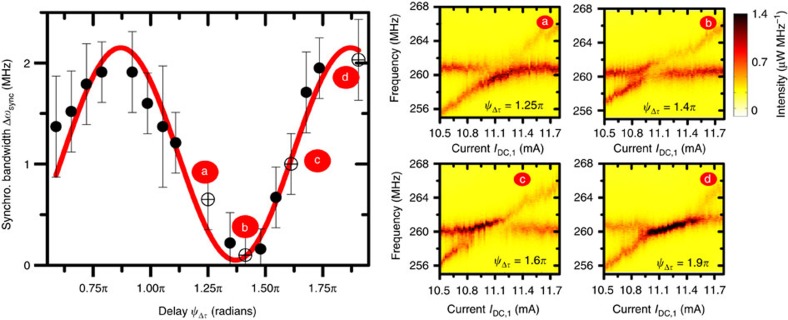
Evolution of the synchronization bandwidth of the two mutually synchronized oscillators depending on the delay constant (associated to the length of the delay line). The DC current applied to STO 1 is +10.6 mA and the one applied to STO 2 is swept. Colour maps of the power spectral density as a function of frequency and *I*_DC,1_ at different delays: intermediate synchronization bandwidth (a) for *ψ*_Δ*τ*_=1.25*π*, minimum (b) for *ψ*_Δ*τ*_=1.4*π*, intermediate (c) for *ψ*_Δ*τ*_=1.6*π* and maximum (d) for *ψ*_Δ*τ*_=1.9*π*. The error bars correspond to the limits of the current zone for which phase slips (linewidth enhancements) appears and disappear.

**Figure 5:**
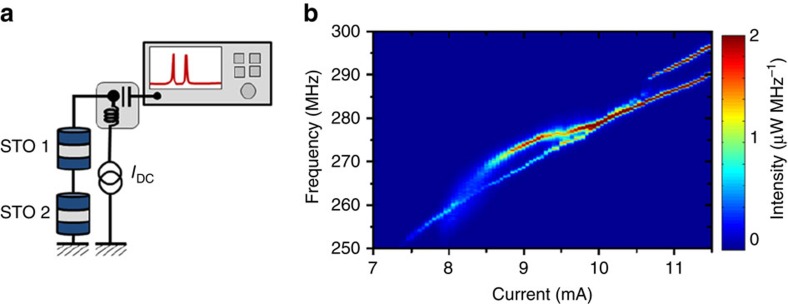
Electrical synchronization of two spin torque oscillators connected directly in series. (**a**) Electrical circuit for the synchronization of two nano-oscillators connected in series with a single DC current source. The two oscillators are connected by a gold wire bonding and the length of cable between the two oscillators is of a few mm. (**b**) Power spectral density of the two nano-oscillators depending on the applied DC current at *H*_perp_=200 kA m^−A^. (The sample diameter is 300 nm).

The measurements shown in Figs 1, 2 and 3 have been performed using an optimized delay between the two STOs. Here after we present how this delay is a crucial parameter to control the synchronized state. Indeed, the synchronization bandwidth Δ*ω*_sync_, that is, the frequency range in which the two STOs have a common frequency, is predicted to depend not only on the strength of the synchronizing force *F*_e_, but also on the phase difference between the two STOs^20^,^25^:


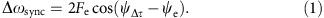


with 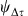 the delay introduced by the delay line^26^,^27^, 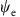 the intrinsic phase shift between the two STOs^23^. Our approach has been to introduce an electrical delay line (Fig. 1a) that permits to tune the total phase difference through the control of the delay constant 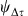. In Fig. 4, we present the evolution of the mutual synchronization bandwidth as a function of the delay. There, we clearly observe a π-periodic oscillation of the synchronization bandwidth with the delay constant. Indeed, by selecting the proper electrical delay 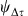, we can either maximize (Fig. 4d) or minimize (Fig. 4b) the synchronization bandwidth. We note that the maximum amplitude of the synchronization bandwidth does not exceed 2 MHz only because of the modest amplitude of the synchronizing force *F*_e_ for the input parameters (DC current, magnetic field) used for these measurements (Supplementary Note 3). We can obtain larger synchronization bandwidths (around 10 MHz) by connecting two STOs directly in series (without the use of a power splitter) as we show latter in Fig. 5. Moreover, we want to remind that the synchronization bandwidth is proportional to the emitted microwave power of the STOs. For this study, the average emitted power of each STOs when they are not in interaction is about 400 nW (Fig. 2b) but much larger power could be reached with other types of STOs, notably using FeB based MTJs (>2 μW)^27^.

Another important result from Fig. 4 is that it shows maxima (respectively minima) of the synchronizing bandwidth for delays 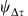 around 9*π*/10 (modulo *π*) (respectively 2π/5 (modulo *π*)). As we will show in the following, this observation is essential to determine the actual origin of the synchronizing force.

The implementation of a delay line between the two STOs allows the control of the frequency of the mutually synchronized state *ω*_s_. The frequency in the synchronized state thus does not depend only on the eigenfrequency of each oscillator but also on the frequency detuning Δ*ω*=*ω*_STO,2_−*ω*_STO,1_ between the two STOs^7^:


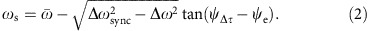


From equation (2), we can conclude that the synchronized frequency *ω*_s_ equals the average frequency 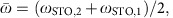 (with *ω*_STO,2_ and *ω*_STO,1_ the frequencies of the non-interacting STOs) when the phase difference 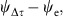 is close to zero (mod π). This condition is similar to the one to get a maximum synchronization bandwidth (see equation (1)). In Fig. 4d, we show that the experimental frequency of the synchronized state is indeed equal to the average frequency of the two STOs in the case of a maximum synchronization bandwidth. For a lower synchronization bandwidth, that is, 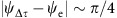, the frequency in the synchronized regime differs from 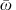, the average frequency. Depending on the sign of the tangent term in equation (2), the frequency can be either larger or smaller than 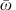. This is confirmed experimentally as displayed in Figs 4a-4c where the synchronized frequency is either higher (for 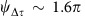 rad) or lower (for 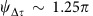 rad) than the average frequency for intermediate synchronization bandwidths. Note that our analysis is performed in the middle of the synchronization bandwidth (Δ*ω*∼0) where there is no frequency pulling. The microwave features of two synchronized STOs thus strongly depends on the delay between them, which could be of great interest for tuning the filtering functionality of arrays of synchronized STOs^18^ or in the perspective for development of bio-inspired associative memories^12^,^28^,^29^. In fact, it is today accepted that neurons in the brain behave like nonlinear oscillators that are coupled through synapses allowing the communication between them. The activities of the synapses control the coupling weights between them and the learning capabilities of the brain. In the case of STO networks, we demonstrate here that a global and long-range electrical coupling leads to a similar tunable coupling. It thus becomes realistic that some of the brain functionalities might be reproduced soon using spin-torque oscillators connected through magnetic domain walls based memristors^12^,^30^ for tuning the coupling strength between STOs.

### Field-like torque drives the phase shift between synchronized oscillators

From these aforediscussed results, we expect a maximum synchronization bandwidth for zero electrical delay between the two STOs. Such a feature raises the prospect of the synchronization of multiple STOs without the necessity of adding a large length of microwave cable between each oscillator and so to avoid detrimental constraints for microwave frequency applications^31^. Indeed, the synchronization bandwidth (equation (2)) of two STOs depends drastically on their intrinsic phase shift 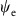, which corresponds to the phase shift for zero electrical delay 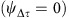 and is expressed for a vortex-based STO as:


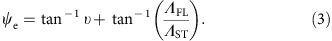


The intrinsic phase shift 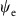 is not only related to the term associated to the nonlinear oscillator parameter *υ*^20^ (as it is often the case for other types of oscillators^7^,^13^,^32^) but also to the ratio of the two components of spin torque responsible for the synchronization, that is, the Slonczewski like Torque (*Λ*_SL_) and the field-like (FL) Torque (*Λ*_FL_)^33^. Through the analysis of time domain measurements, we can experimentally determine the nonlinear parameter *ν* (Supplementary Note 1) resulting in tan^−1^ *υ*∼2*π*/5 in equation (3). Moreover, we know from our previous studies on vortex-based STOs^23^,^34^ that the FL torque is large compared to the SL Torque leading to 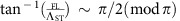. Thus, we expect a non-zero synchronization bandwidth for two STOs directly connected in parallel or in series, which is not possible for spin wave coupled STOs (for which, 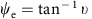 (refs 1, 2, 3)).

According to equation (2), we expect maxima of the synchronization bandwidth for 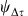 close to 9*π/10* (mod *π*) which is in excellent agreement with the results represented in Fig. 4. This robust observation indicates the possibility to obtain a large synchronization bandwidth Δ*ω*_sync_ for 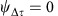, that is, without a delay line.

To confirm this assertion, we have connected two STOs (300 nm diameter) directly in series with one single current source as represented in Fig. 5. For *I*_DC_ between 9 and 9.8 mA, the two STOs are not synchronized. The STO with the highest frequency adapts its frequency to the second one leading to a frequency pulling. Then, the two oscillators synchronize for *I*_DC_ between +9.8 and +11 mA and desynchronize for larger current values. The frequency of the synchronized state is always close to the frequency of one of the two STOs. We can correlate this behavior with the fact that the microwave properties of each individual STO are far from being identical like the ones used in the previous measurements.

We emphasize that the synchronization bandwidth reaches about 10 MHz and a current range of more than 1 mA (close to the spin-wave coupling presented in ref. 3). Note that the synchronization bandwidth is here about five times larger than the maximum one for the two STOs connected in parallel (as seen in Figs 1, 2, 3, 4). This difference can be mainly and simply explained because we do not use here the −6 dBm power splitter to connect the two STOs as in the parallel case. Finally, we would like to stress that this observation confirms that we are close an optimized (and maximum) synchronization bandwidth at zero electrical delay. This result also demonstrates that the integration of more than two STOs won't present any additional difficulties to succeed the electrical synchronization of STO arrays in a near future.

## Discussion

This mutual synchronization of two spin torque oscillators for zero electrical delay is a remarkable feature, which highlights the crucial role of the Field-like Torque in the synchronization process of vortex based spin-torque oscillators (STOs). This is a promising and unexpected result as all the pioneering theoretical studies on uniformed based spin-torque oscillators have considered the Field-like torque to be negligible in the synchronization process^7^,^31^,^32^. Interestingly, one can notice that the efficiency of the different locking torques is also known to change as a function of the bias voltage^35^,^36^ which provides an additional parameter to optimize the intrinsic phase shift 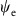. The electrical synchronization of spin-torque oscillators with zero phase difference, combined with the drastic improvement of the microwave-features (linewidth and power) in the synchronized state, marks an important milestone towards a new generation of microwave-devices based on spin-torque oscillators. Furthermore, the full control of the electrical synchronization of two spin-torque oscillators, both externally with an electronic delay and intrinsically through the ratio of the spin transfer torques, opens among others the perspective of spin-torque oscillator networks mimicking some of the basic brain functionalities such as patterned recognition and classification^12^,^16^,^17^.

## Methods

### Sample description

The data presented is for circular-shape magnetic tunnel junctions (MTJ) with a nominal diameter of 300 nm. The multilayer stacking of each MTJ is composed of a double vortex spin-valve on top of a magnetic tunnel junction: Synthetic antiferromagnet (SAF)/MgO (1.075)/NiFe (6)/Cu(9.5)/NiFe (20) (with thickness in nm). The pinned SAF layer is a PtMn (15)/CoFe (2.5)/Ru (0.85)/CoFeB (3) multilayer. The two NiFe layers have a magnetic vortex as ground state. The GMR ratio of the Cu based spin-valve is about 2% whereas the TMR ratio of the MgO based MTJ is about 70% at room temperature and low bias. Therefore, the output power that is detected on the spectrum analyzer is predominantly arising with the vortex dynamics in the thin NiFe layer that is close to the MgO barrier. More detail about these double vortex based STOs can be found in ref. 22. Note that the power of the coupled vortex STOs studied here is larger than the one in ref. 22 because in this latter study, the STOs have the thin excited vortex layer not in contact with the MgO but on the top layer of the spin-valve.

### Description of the experimental set-up

We source individually the two STOs with two independent DC sources (Fig. 1). To study the electrical mutual synchronization, the two STOs are connected using conventional microwave cables and bias tees. Moreover, to tune (manually) the delay time between the two STOs, we have also introduced an electrical delay line as shown in Fig. 1, that allows us to vary the the delay time. The detection of the total emitted signal is obtained using a spectrum analyzer connected to the electrical circuit through a −6 dBm power splitter.

### Preparation of the STOs magnetization state

To prepare magnetically the state in the two STOs, we perform a field cycling before the electrical measurements to initialize each double vortex system in parallel chiralities and antiparallel cores polarities, which permits to have sustained oscillations without any applied magnetic field. In fact, we first apply a large perpendicular magnetic field to impose parallel core polarities and then we reverse the field until the core polarity of the thinner NiFe layer eventually reverses. In our convention, a positive current corresponds to electrons flowing from the thin to the thick NiFe layer, resulting in the spin transfer dynamics of the coupled vortex mode that is mainly located in the thin NiFe layer. See ref. 22 for details about the characteristic of the different coupled modes and the symmetry of the spin transfer forces.

### Data availability

The data underlying the present work are available on request from the corresponding authors.

## Additional information

**How to cite this article:** Lebrun, R. *et al*. Mutual synchronization of spin torque nano-oscillators through a long-range and tunable electrical coupling scheme. *Nat. Commun.* **8,** 15825 doi: 10.1038/ncomms15825 (2017).

**Publisher's note:** Springer Nature remains neutral with regard to jurisdictional claims in published maps and institutional affiliations.

## Supplementary Material

Supplementary Information Supplementary Figure, Supplementary Notes and Supplementary References
