# Supplementary material for: Mutual synchronization of spin torque nano-oscillators through a long-range and tunable electrical coupling scheme
Source: Nat Commun. 2017 Jun 12;8:15825. doi: 10.1038/ncomms15825 (PMC5472782; doi:10.1038/ncomms15825)
Supplement: Supplementary Information — Supplementary Figure, Supplementary Notes and Supplementary References [file ncomms15825-s1.pdf]

Supplement note 1: Characteristics of the non-interacting spin torque oscillators (STOs)

Supplementary Figure 1 shows the properties of each non-interacting STOs. In the measurements presented in the main text, the dc-current $I_{DC,2}$ of the oscillator n°2 (STO 2) is fixed to $I_{DC,2}$ = +10.6 mA, while the dc-current of the oscillator n°1 (STO 1) is swept from +10.5 to +11.8 mA. For these conditions, the properties of the rf emitted signal are similar i.e. the spectral linewidth (STO 2 ≈ 1.15 MHz, STO 1 ≈ 1-1.25 MHz), the emitted power (STO 2 ≈ 0.44 μW, STO 1 ≈ 0.35-0.52 μW) and their nonlinear parameter ν (STO 2 ≈ 3.4, STO 1 ≈ 3-4.8). Having chosen these dc current values permits us to stand in the hypothesis of "almost identical" oscillators[1], a condition that is particularly true in the middle of the synchronization bandwidth.

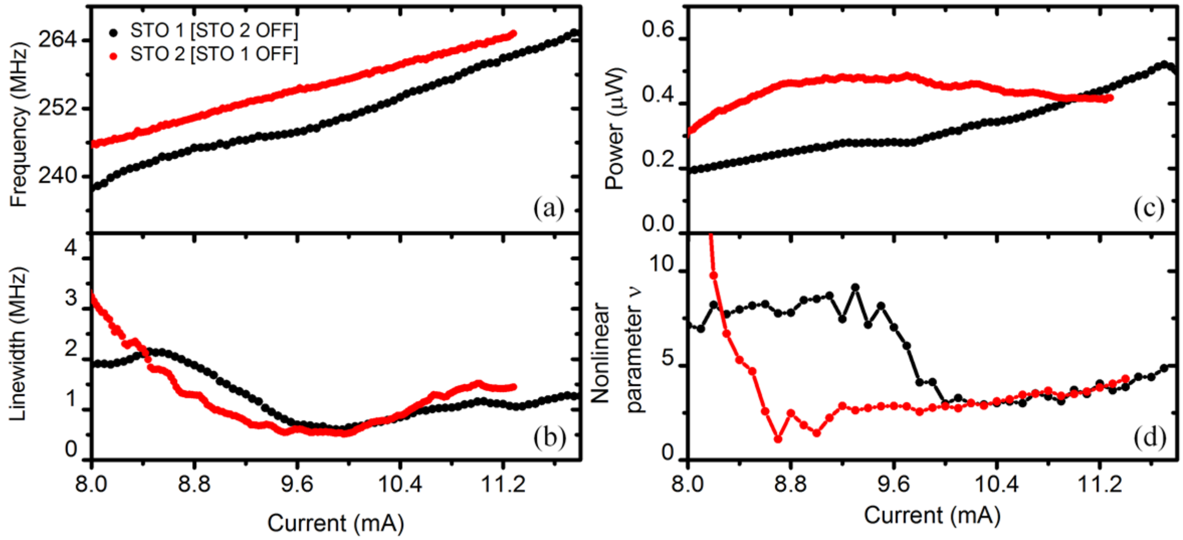

Supplementary Figure 1: Properties of the non-interacting STOs. Frequency (a), Linewidth (b), Power (c) and nonlinear parameter υ (d) for each non interacting oscillator (STO 1 and STO 2) as a function of I_{DC} in their respective current source. These measurements have been obtained at room temperature and zero field.

Supplement note 2: Characteristic of the phase noise in the synchronized state

In the optimized synchronized state, corresponding to the largest synchronized bandwidth, i.e. $\psi_{\Delta\tau} - \psi_e \approx n\pi\ [\pi]$, the phase sum and difference are defined by the following system of **Supplementary Equation 1** (see Ref [2]):

$$\begin{cases} \dfrac{d\Phi}{dt} = -2\overline{\omega} + \xi_1(t) + \xi_2(t) \quad (1) \\ \dfrac{d\psi}{dt} = -\Delta\omega - 2F_e \sin\psi + \xi_1(t) - \xi_2(t) \quad (2) \end{cases}$$

where $\xi_1(t)$ and $\xi_2(t)$ are the white noise source in each STO, $2\overline{\omega}$ the sum of the two STO frequencies $\Delta\omega$ the frequency detuning and F_e the strength of the locking force. For almost identical oscillators, the intensities of noise are equivalent (leading to similar spectral linewidths) and described by **Supplementary Equation 3**:

$$\langle \xi_1(t) | \xi_2(t') \rangle = 2\sigma_0^2 \delta_{1,2} \delta(t - t') \qquad (3)$$

Contrary to the phase difference, the phase sum Φ has no retroaction process. Thus, the diffusion of the phase sum turns out to be much larger than the diffusion of the phase difference with a diffusion constant $D_\Phi = 2\sigma_0^2 \gg D_\psi$. Given that the phases of each synchronized oscillator are $\phi_1 = (\Phi + \psi)/2$ and $\phi_2 = (\Phi - \psi)/2$, their diffusion constants are then $D_{s,1} = D_{s,2} = \sigma_0^2$. Note that when the two STOs are not interacting, their diffusion constants are $D_1 = D_2 = 2\sigma_0^2$. We thus deduce that the phase noise of the synchronized STOs should decrease by a factor 2 compared to the value when they are independent.

Supplement note 3: Estimation of the synchronization bandwidth

As mentioned earlier, the two STOs are almost identical. In this case, the rf-current emitted by one STO that will drive the second oscillator can be estimated by **Supplementary Equation 3**:

$$J_{rf,STO} = \frac{I_{rf,STO}}{\pi R^2} = \frac{\sqrt{P_{int}/R_{STO}}}{\pi R^2} \qquad (3)$$

with R the dot radius of the STO, R_{STO} the STO resistance and P_{int} the power emitted by the STO. In our experimental setup, the presence of a power divider used to detect the emitted power on a spectrum analyzer results in a decrease by 6 dBm of the effective synchronizing signal that is applied on each oscillator. The impedance mismatch between the circuit resistance (50 Ω) and the oscillator resistance (32 Ω) is also contributing to the decrease of the actual synchronizing rf-current. We estimate for this effective synchronizing current $I_{rf,eff} = (1 - R_{re}) I_{rf,STO}/2$ with R_{re} = (50-32)/(50+32).

The synchronization bandwidth is then expressed in the best conditions, i.e for $\psi_{\Delta\tau} - \psi_e = n\pi\ [\pi]$, by **Supplementary Equation 5**:

$$\Delta\omega_{sync} = 2\frac{\sqrt{1+\upsilon^2} * \sqrt{\Lambda_{SL}^2 + \Lambda_{FL}^2}}{\sqrt{p_0}} J_{rf,eff} \ (4)$$

and the normalized power by **Supplementary Equation 5** [3]:

$$p_0 = \frac{18 P_{int}}{R_{load}} \left(\frac{R_{load} + R_{STO}}{I_{dc} \Delta R_{TMR}}\right)^2 \ (5)$$

For the presented measurements, $I_{rf,STO} = 0.5\ mA$, $R_{STO} = 32\ \Omega$, $\upsilon = 3.4 - 3.7$, $p_0 = 0.21$ ($\Delta R_{TMR}(I_{dc})$ ~ 14 Ω, R_{load} = 50 Ω, R_{STO} = 30 Ω, I_{dc} = 11 mA) , R = 150 nm, and Λ_{FL}^2 and Λ_{SL}^2 are taken from Reference [4]. Injecting these parameters, we deduce $\Delta\omega_{sync}$~ $1.6 - 2.1$ MHz. Note that this value is in excellent agreement with our experimental observations (2.2 MHz).

Supplement References

1. Slavin, A. & Tiberkevich, V. Nonlinear Auto-Oscillator Theory of Microwave Generation by Spin-Polarized Current. IEEE Transactions on Magnetics 45, 1875–1918 (2009).
2. Pikovsky, A., Rosenblum, M. & Kurths, J. A universal concept in nonlinear sciences, Cambridge University Press (2001).
3. Grimaldi, E. et al. Response to noise of a vortex based spin transfer nano-oscillator. Phys. Rev. B 89, 104404 (2014).
4. Jenkins, A. S. et al. Spin-torque resonant expulsion of the vortex core for an efficient radiofrequency detection scheme. Nature Nanotechnology (2016). doi:10.1038/nnano.2015.295
